# How should we teach homeostasis? Filling the gaps and envisioning the future

**DOI:** 10.3389/fphys.2026.1784015

**Published:** 2026-03-02

**Authors:** Serena Y. Kuang, Akshata R. Naik

**Affiliations:** Department of Foundational Medical Studies, Oakland University William Beaumont School of Medicine, Rochester, MI, United States

**Keywords:** emergent property, homeostasis, homeostatic tendency, integrative physiology, internal environment, metabolomics, oscillation, steady state

## Abstract

This article provides a conceptual and pedagogical analysis aimed at advancing homeostasis education within integrative physiology. Although homeostasis was originally formulated to describe the stability of the internal environment (IE), educators have traditionally emphasized the regulation of individual parameters such as body temperature, blood glucose, and blood pressure. This emphasis reflects historical methodological limitations rather than the full conceptual scope intended by Claude Bernard and Walter Cannon. Building on conceptual developments articulated in Kuang (2023), the present analysis examines how homeostasis education can be refined and reoriented by clarifying several foundational conceptual distinctions. In particular, it addresses persistent gaps in current teaching, including common ambiguities related to the concepts of steady state, dynamic equilibrium, constancy, and stability and builds upon the distinction between parameter homeostasis and, IE homeostasis to strengthen its pedagogical and conceptual application. At the organism level, the article addresses how, IE homeostasis can be appropriately interpreted and taught, highlighting its emergent nature and the role of modern integrative measurements, including multi-omics approaches, in supporting a system-level understanding of homeostasis. Overall, the analysis supports the following central conclusions: (1) Parameter homeostasis refers to the relatively stable oscillation of the parameter value; (2) IE homeostasis constitutes a high-level emergent property of the organism; (4) modern integrative measurement approaches make it possible to study the IE as a whole, providing system-level readouts relevant to IE homeostasis; and (4) the conceptual scope of homeostasis or homeostatic tendency, is expanded in two directions: across health, subhealth, disease, and psychological steady states, and toward an explicit recognition of the constitutive role of organism–environment interactions in shaping internal stability. In sum, this article envisions an integrated direction for homeostasis education that moves beyond parameter-centric approaches and supports a coherent understanding of organismal regulation, adaptation, and resilience across diverse conditions.

## 1 Introduction

The concept of internal stability within the body of an organism dates back to the 19th century. Claude Bernard famously proclaimed that “the fixity of the milieu intérieur is the condition of a free, independent life” (Bernard, 1865; Bernard, 1879; Cooper, 2008) and described the internal environment (IE) in which cells and tissues live in relative independence from external conditions. This statement articulated, in a systematic and influential manner, the idea that life depends on the relative constancy of the IE.

In the early 20th century, Walter Cannon (1926), Walter Cannon (1932) extended Bernard’s insight by emphasizing that higher organisms can maintain such internal stability through coordinated self-regulatory mechanisms despite external disturbances and conditions that threaten physiological equilibrium. Cannon introduced the term *homeostasis* in 1926 and later provided a systematic account in his classic book *The Wisdom of the Body* (1932). The term was soon widely accepted and became the central organizing concept of physiology. Together, the insights of Bernard and Cannon laid a visionary foundation that continues to shape modern physiology.

Despite its enduring influence, the term *homeostasis* has carried an inherent ambiguity since its inception. It remains unclear whether Cannon referred primarily to the phenomenon of a relatively stable IE, the regulatory mechanisms that achieve this stability, or both. In practice, Cannon (1932) employed both ways of description, at times referring to the stable state and at other times to the processes that sustain it, without explicitly delineating them.

This ambiguity persists in contemporary definitions. As summarized in Kuang (2023), definitions of homeostasis across textbooks and educational resources variously define homeostasis as a phenomenon of internal stability, the mechanisms that maintain it, an ability of biological systems, or a stabilizing tendency. These variations do not merely reflect semantic differences; they point to conceptually distinct attributes operating at different explanatory levels.

Recognizing this multiplicity, rather than forcing homeostasis into a single, monolithic definition, motivates the need for a structured, multi-dimensional conceptual framework, as developed in Kuang (2023). Building on that framework, the present article aims to: first, briefly highlight key developments in the concept of homeostasis articulated in Kuang (2023); second, address persistent gaps in conventional teaching; third, consider future directions for homeostasis education from the perspectives of integrative physiology, and fourth, provide a brief pedagogical scaffolding to facilitate homeostasis education.

## 2 New developments in the concept of homeostasis Built on Bernard’s and Cannon’s legacies

Several new developments to understand homeostasis and unravel its complexity in Kuang (2023) are highlighted as follows.

### 2.1 The concept of homeostasis needs to be understood from multiple dimensions

Based on the introduction above, what is commonly referred to as “homeostasis” involves several closely related aspects. The observable stability of the IE reflects the surface-level phenomenon, the regulatory processes that produce this stability represent the underlying mechanisms, and the outcome of these processes corresponds to what Bernard described as a free and relatively independent life. These are not competing definitions, but different dimensions of the same complex concept.

Importantly, homeostasis cannot be adequately understood by considering these aspects in isolation. As a holistic property of the organism, homeostasis necessarily depends on the internal functional organization of the body, in which bodily functions are interconnected and arranged in a hierarchy. This internal, interconnected, hierarchical organization is therefore fundamental to homeostasis, constituting an essential dimension of it.

Together, these considerations support a four-dimensional understanding of homeostasis (Kuang, 2023): internal functional organization (first dimension), functional manifestation (second dimension), regulatory mechanisms (third dimension), and outcome (effect or consequence, fourth dimension). Built upon the core insights of Bernard and Cannon, this framework integrates different perspectives on homeostasis and helps clarify the ambiguity in its definition.

### 2.2 Understanding homeostasis in terms of homeostatic tendency

Through logical and conceptual analysis, Kuang (2023) proposed that homeostasis may be understood in terms of *homeostatic tendency*, rather than as a homeostatic phenomenon or the regulatory mechanisms underlying it. As a tendency, homeostasis does not denote a fixed state, nor does it reduce to specific control processes; instead, it refers to a dynamic property of the IE that is oriented to achieve relative internal stability through two types of complementary regulations as follows.

On the one hand, homeostatic tendency may be expressed through stabilizing regulation, by maintaining or restoring a previous steady state of the IE when perturbations are transient or limited. When stabilizing mechanisms are insufficient to preserve the steady state in the face of sustained internal or external challenges, homeostatic tendency is expressed through adaptive regulation, leading to the establishment of a new, variable steady state. Once the new steady state is achieved, stabilizing mechanisms again predominate to maintain this new condition. If environmental demands continue to change and the new steady state can no longer be sustained, further adaptive transitions may occur to establish a newer, variable steady state. In this sense, homeostasis is neither static nor episodic, but a continuous, layered process that integrates stability and adaptability over time (Kuang, 2023). Here, “homeostatic tendency” is not proposed as a replacement term, but as an alternative term that foregrounds its dynamic, layered, and adaptive character and facilitates its understanding.

Historically, however, this dual nature of homeostasis has been obscured in physiology education. As Carpenter (2004) pointed out, the concept has often been reduced in textbooks to the point of being treated as nearly equivalent to negative feedback regulation around a fixed set point. In contrast, Cannon’s original writings on homeostasis emphasized the capacity of organisms to maintain relative internal stability under changing external conditions (Cannon, 1926; 1932). Such adaptability is not optional. It is an inherent requirement for internal stability in a changing environment.

Moreover, Cannon’s analyses of biological regulation provided not a single regulatory template, but a variety of biological exemplars that later inspired the development of control theory (Carpenter, 2004). Regulatory strategies now formalized in control theory, including internal feedback, feedforward control, predictive regulation, and hierarchical organization, can all be traced back to the biological examples Cannon described carefully. Control theory is taken as a framework that emphasizes regulation under changing conditions, this historical continuity supports the view that adaptability was already implicit in Cannon’s original conception of homeostasis. Nevertheless, in recent textbook presentations of homeostasis, adaptation has often been overlooked.

In response to these limitations, several concepts have been introduced to supplement homeostasis, including allostasis (Sterling and Eyer, 1988; McEwen and Stellar, 1993; McEwen and Wingfield, 2003), predictive homeostasis or predictive regulation (Sterling, 2012; Schulkin and Sterling, 2019), and adaptive homeostasis (Davies, 2016). These developments reflect both the longstanding ambiguity surrounding the concept of homeostasis and the growing recognition of the complexity of physiological regulation. A more effective approach is to clarify the original concept of homeostasis by explicitly articulating its dynamic and multidimensional character.

Viewed from this perspective, homeostasis, or homeostatic tendency, can be understood as the unity of two inseparable yet complementary aspects: stability and adaptability (Kuang, 2023). Such a unified framework has the potential to serve as the true central organizing concept or framework of physiology, extending well beyond the oversimplified, single-dimensional definitions that dominate current teaching resources. Within this framework, newer terms are naturally encompassed rather than treated as competing alternatives, thereby preserving conceptual coherence.

Here, we further abstract the two types of complementary regulatory mechanisms as stabilizing and adapting modes. This terminological shift reflects a conceptual refinement rather than a change in meaning: each physiological parameter can operate in either a stabilizing or an adapting mode, whereas the concrete mechanisms through which these modes are implemented are parameter-specific and may differ substantially across systems.

### 2.3 Homeostasis of the IE and homeostasis of a parameter should be distinguished

Both Bernard and Cannon noted the importance of IE stability, but given the limitations of the methods, they could not actually study the stability of the IE as a whole. What they studied instead was the regulation of individual parameters, such as blood glucose, electrolytes, pH, or body temperature. However, a collection of parameters is not the same as the IE itself.

As mentioned in Kuang (2023), this also shaped the way physiology education developed. In textbooks and classrooms, the concept of homeostasis is often introduced as it refers to the stability of the IE. However, because the complexity of the IE is not always emphasized, the discussion quickly deviates into discussing the homeostasis of individual parameters without a clear distinction or transition. As a result, students are left confused regarding whether homeostasis is about the stability of the IE as a whole or about the regulation of individual parameters and their fundamental differences and interdependence. Physiology education needs to make this distinction clear by properly addressing the similarities and differences between the two. This logical step was missing in the past, but it is essential for both conceptual clarity and effective teaching.

The four dimensions of homeostasis and the concept of homeostatic tendency mentioned above apply to both the homeostasis of the IE and the homeostasis of a parameter.

Building on Kuang (2023), the present article focuses on how to strengthen physiology education in the area of homeostasis of a parameter, since this is where most of the teaching actually happens and also where major misconceptions exist. We then turn to the future and discuss how new technologies, such as multi-omics, and especially metabolomics, may help us to study and teach the homeostasis of the IE itself.

### 2.4 Relativity is the inherent characteristic of homeostasis

Readers are encouraged to refer to Kuang (2023), in which a dedicated subsection (Section 3.3) systematically examined the relativity of homeostasis. In brief, homeostasis does not imply an absolute or context-free internal stability. Rather, any steady state—whether of an individual parameter or of the IE—is meaningful only with respect to a specific temporal and spatial reference, such as the time scale of adaptation or the environmental and physiological context. Transitions among modifiable steady states therefore reflect reference-dependent adaptations, not departures from homeostasis.

As an inherent characteristic and a defining feature of homeostasis, this relativity should be explicitly emphasized in physiology education. However, among the 21 definitions of homeostasis surveyed in the supplemental table of Kuang (2023), only three explicitly acknowledged the relative nature of stability. This omission highlights a conceptual insufficiency in current teaching resources and underscores the need to strengthen how the relativity of homeostasis is presented and taught in the future.

Framing relativity as a new development may overstate its novelty, since the relative nature of homeostasis has long been implicit in physiological practice. The contribution here lies in making this implicit feature explicit and treating relativity as an indispensable component of how homeostasis should be understood and taught. In this sense, teaching homeostasis without relativity risks reducing a fundamentally dynamic process to an oversimplified and static account.

The relativity of homeostasis is straightforward to illustrate at the level of individual parameters (see Section 3), whereas the relativity of the IE’s homeostasis is more complex, yet more revealing of why homeostasis is inherently relative (see Section 4).

## 3 Filling the gaps in teaching the homeostasis of a parameter

In current education, teaching a negative feedback loop for the homeostasis of individual parameters is well-covered. We address gaps in this teaching by clarifying the meanings of several key terms describing the homeostasis of a parameter. We also suggest how physiology education can present a more accurate and unified view of homeostasis of a parameter.

### 3.1 What do we mean by *steady state* and *dynamic equilibrium*?

When Cannon coined the word *homeostasis*, he was very deliberate. *Homeo* means “similar” or “unchanging,” and *stasis* means “standing” or “condition.” Put together, the word conveys the idea of a *steady state*. We might also call this a dynamic equilibrium, although Cannon preferred to avoid the term “equilibrium”^1^.

In most teaching resources, the description of *steady state* leads to a common misunderstanding of a constant, unchanging value of a parameter, such as body temperature fixed at 37 °C. However, the parameter value fluctuates within limits around a set point, and continuous regulatory processes supported by energy expenditure maintain this condition. It is important to educate learners that a steady state or a dynamic equilibrium does not imply that the parameter value itself is steady; rather, the pattern by which that value fluctuates over time is steady. Only by making the meaning of steady state explicit can students understand why the homeostasis of a parameter is inherently dynamic rather than static.

### 3.2 “Stable oscillation”: an underused but accurate term for what is in *steady state*

**FIGURE 1:**
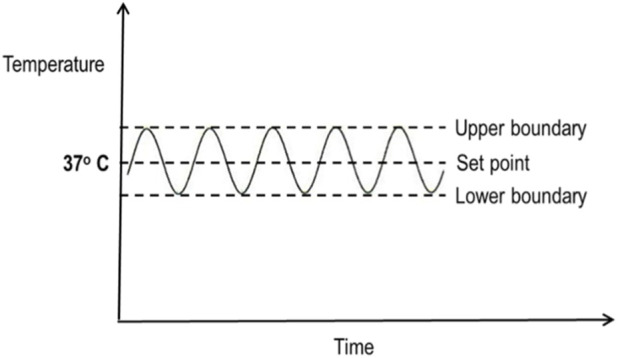
Homeostasis of the body temperature. At the parameter level, the stable feature is the oscillatory pattern of the parameter (body temperature) around a set point, rather than the absolute temperature value itself.

To clarify that *steady state* does not apply to the absolute value of a parameter, but to its regulated behavior over time, we routinely use the following simple multiple-choice question in teaching (Figure 1).

Question 1 What is *in steady state*? Select all that apply.

**(A)** The value of body temperature (No).

**(B)** The set point of body temperature (Yes).

**(C)** The upper and lower limits of body temperature (Yes).

**(D)** The oscillation of body temperature (Yes).

This question allows students to see that *steady state* does not refer to the value of the parameter itself, but to the oscillation of the value around a set point. In this context, the stability of the set point, the persistence of upper and lower limits, and the continuous fluctuation of the parameter value converge on the concept of parameter homeostasis. Accordingly, it is not the parameter value itself but its oscillation that is stable; the homeostasis of a parameter is therefore most accurately described as a relatively stable oscillation.

Despite its precision, the concept of stable oscillation is rarely used in teaching, leading to persistent conceptual ambiguity. When explicitly identified as the defining feature, dynamic equilibrium becomes clear as an alternative term for stable oscillation.

### 3.3 It is necessary to rethink the terminologies “fixity”, “constancy”, and “stability”

From Bernard to Cannon, terms such as *fixity*, *constancy*, and *stability* played a central role in the early framing of the stable, IE and physiological regulation. In their historical context, Bernard’s emphasis on the “constancy of the milieu intérieur” (Bernard, 1865; Bernard, 1879) and Cannon’s focus on the “stability of the internal environment” (Cannon, 1926; Cannon, 1932) conveyed a crucial insight: life depends on maintaining internal order despite continuous external perturbations. From a historical perspective, this language was both necessary and illuminating.

However, when these terms are adopted in contemporary physiology teaching without clarification, they invite misinterpretation. Students may interpret constancy as an unchanging value and stability with immobility—interpretations that diverge from Bernard’s and Cannon’s original intent but arise naturally when historical context is no longer explicit.

For this reason, while the historical significance of classical terminology should be acknowledged, its pedagogical limitations must also be recognized. Rather than relying on fixity, constancy, or stability alone, explicitly framing parameter homeostasis in terms of relatively stable oscillation preserves Bernard’s and Cannon’s original insights while avoiding static interpretations.

### 3.4 What if physiological parameters did not oscillate but remained static?

Understanding that *stable oscillation* is the accurate phrase for what is in a steady state is an important first step. To reinforce this understanding, students should also be guided to ask a deeper question: What if all the physiological parameters did not oscillate at all, but instead remained completely static?

This kind of question seems not to be commonly asked in current physiology education, yet it is crucial. The answer is straightforward but profound: if parameters were truly fixed and unchanging, the organism would lose its adaptability. Without oscillation, the system would have no flexibility, no capacity to adjust to perturbations, and ultimately no ability to survive.

**FIGURE 2:**
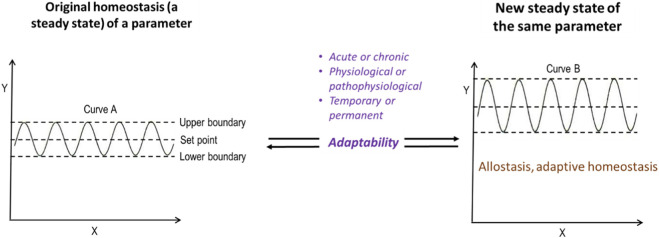
The unity of stabilizing and adapting modes in the homeostasis of a parameter. When a parameter is in steady state oscillation (SS1), the stabilizing mode maintains or restores this oscillatory pattern of the parameter against small perturbations, rather than preserving an absolute parameter value. If larger perturbations occur and SS1 cannot be maintained, the adapting mode shifts the system toward a new steady state oscillation (SS2) with a modified set point. Once SS2 is established, the stabilizing mode again takes over. This process may repeat (SS3, SS4, etc.), showing that parameter homeostasis is not limited to stability or adaptability alone, but reflects the unity of both. Increasing the range of oscillation is hypothetical because it may be possible, but the literature lacks data to address this scenario, leaving it a topic for future research. Steady state oscillation and stable oscillation have the same meaning.

*Oscillation* of a parameter value, therefore, is not a minor detail but the very feature that connects stability to adaptability. It shows students why homeostasis of a parameter cannot be equated with simple constancy. By asking and answering this counterfactual question, teaching can reinforce the central role of *stable oscillation* of a parameter, ensuring that students grasp both its precision as a term and its essential biological meaning. Figure 2 illustrates how the oscillation of a parameter adapts to the environmental changes to achieve a new steady state, or in other words, a new stable oscillation by changing the set point of the oscillation.

For clarity, in this article the notation SS1, SS2, and SS3 (and higher-order extensions) is used exclusively to describe layered steady states of individual physiological parameters (Kuang, 2023). Briefly, this relatively stable oscillation can be preserved in two complementary ways: either by stabilizing the existing oscillation around an unchanged set point, or by adapting to a new set point and establishing a new relatively stable oscillation. In this sense, the homeostasis of a parameter reflects a homeostatic tendency expressed through the regulation of oscillatory behavior.

### 3.5 The relativity of parameter homeostasis

The sequence of layered steady states (Figure 2) provides a concrete example of the relativity of parameter homeostasis. What constitutes a relatively stable oscillation (SS1, SS2, SS3, etc.) depends on the prevailing regulatory context and cannot be reduced to a single, fixed set point. In this sense, parameter homeostasis is maintained through regulated transitions among modifiable steady states and thus reflects the expression of homeostatic tendency at the level of individual physiological parameters.

## 4 Looking forward: teaching the homeostasis of the internal environment (IE)

In this section, the term *homeostasis* refers specifically to the homeostasis of the IE. They are used interchangeably when the context makes the level of organization explicit.

With the advent of modern multi-omics technologies, particularly metabolomics, the holistic study of the IE has become not only possible but increasingly practical. These approaches allow investigators to examine comprehensive metabolic profiles of the IE, including the composition, concentrations, and relative proportions of metabolites, across physiological and pathophysiological processes, psychological distress and mental health states, and diverse environmental and exposomic exposures (Johnson et al., 2016; Walker et al., 2019; Wishart, 2019; Zhu et al., 2022). Rather than focusing on isolated variables, such analyses reveal *patterns* of organization within the IE. These patterns provide a new empirical pathway for understanding how the IE functions as an integrated whole.

In this sense, insights that Bernard and Cannon could only articulate conceptually can now be grounded in measurable data and systematic analysis. This shift, from isolated parameters to system-level patterns emerging at the level of the IE, represents an important epistemological and methodological advance for integrative physiology. It also provides a natural entry point for introducing the concept of *emergent properties*, which is increasingly relevant for physiology education.

From a pedagogical perspective, future teaching of the homeostasis of the IE should emphasize at least four key elements: (1) presenting it as a system-level emergent property at the organism level, (2) explicitly addressing the complexity and heterogeneity of the IE, and the relativity of the IE homeostasis, (3) highlighting the essential technologies that enable the study of IE homeostasis as an integrated whole, and (4) expanding the concept of homeostatic tendency beyond narrowly defined “normal” states.

### 4.1 Teaching the homeostasis of the IE as a unique emergent property at the organism level

An *emergent property* refers to a system-level feature that arises from interactions among components and cannot be fully explained by examining those components in isolation (Emergent property, 2025; Kitano, 2002). From a teaching perspective, the idea can be introduced without formal systems theory, as educators are already familiar with physiological functions that depend on coordinated activity across multiple subsystems rather than any single mechanism.

Viewed in this way, the homeostasis of the IE represents a high-level emergent property at the organism level. It is not the sum of individual parameter regulation, such as temperature, glucose, or blood pressure, but the overall pattern formed by their coordinated behavior. Individual parameters provide partial views of this organization, yet none alone captures how the IE is maintained as an integrated whole.

**FIGURE 3:**
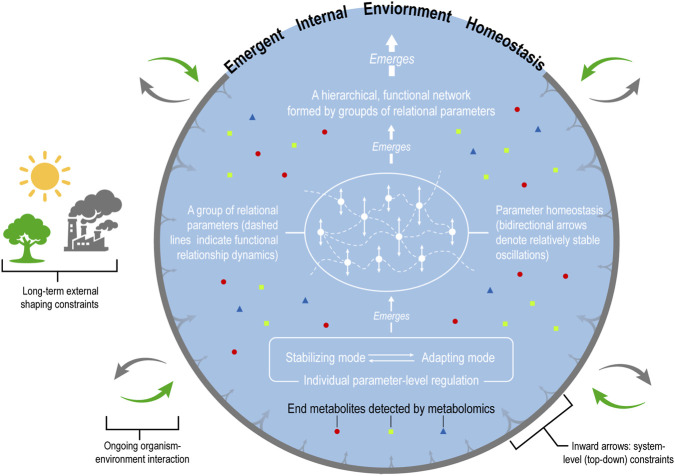
An integrative conceptual framework for emergent internal environment (IE) homeostasis. The diagram conveys four main conceptual elements. (1) It schematically illustrates the progression from regulation at the level of individual parameter homeostasis—implemented through stabilizing and adapting modes, to the organization of dynamic relationships among parameters, and to the formation of a functional hierarchy in which groups of relational parameters integratively give rise to a coherently configured internal environment (IE). (2) Reciprocal to this bottom-up emergence, the, IE as an organized whole exerts top-down, system-level constraints on its constituent subsystems. (3) It highlights metabolomics as a system-level approach for probing end-metabolite profiles that reflect the functional state of the, IE. (4) Interactions between the organism and its environment are explicitly represented as a constitutive factor shaping, IE homeostasis over time.

The IE also shapes how subsystems operate. The global conditions of the IE influence subsystems’ responses to perturbations, the recruitment of regulatory mechanisms, and whether a system returns to a prior steady state or transitions to a new one. In this reciprocal sense, system-level order both arises from and constrains subsystems’ regulatory processes (Figure 3), a principle long recognized in systems physiology (Kitano, 2004; Noble, 2008; Noble, 2011; Woodward, 2020).

### 4.2 Addressing the complexity of the IE and the relativity of IE homeostasis

The complexity of the IE cannot be captured by enumerating individual variables or regulatory pathways. Instead, it requires a framework that organizes its conceptual richness in a coherent way. As developed in Kuang (2023) and introduced earlier in this article, the four dimensions of homeostasis provide such a framework, allowing, IE complexity to be articulated and examined rather than left as a vague intuition.

The relativity of IE homeostasis is more intricate than that of individual parameters and contributes directly to this complexity. IE homeostasis reflects the integrated behavior of multiple interacting parameters, each operating with distinct regulatory dynamics, adaptive capacities, and temporal profiles. As a result, stability and adaptation are not expressed uniformly, but emerge from heterogeneous yet coordinated responses across parameters.

Accordingly, IE homeostasis may be understood in terms of multiple IE overall steady-state patterns or configurations (denoted conceptually as H1, H2, etc.), each defined by its temporal and contextual conditions. Whether a given IE appears stable or adaptive depends on the time scale and observational window: processes that constitute adaptation over longer durations may appear as stability when examined within a short time window. For this reason, any discussion of IE homeostasis necessarily requires explicit temporal framing. A qualitative illustration of this relativity and heterogeneity is provided in the Supplementary Material (Supplementary Figure S1).

### 4.3 Highlighting the essential technology to study homeostasis

From a teaching perspective, the goal is not to introduce technical detail, but to highlight which technologies make organism-level homeostasis empirically accessible. Among these, metabolomics plays a central role by providing an integrative molecular snapshot of the IE, helping students conceptualize the IE as a coherent functional state rather than as a collection of independently regulated parameters. At a broader level, multi-omics approaches support this integrative view by conceptually linking system-wide patterns to underlying regulatory layers.

It is important to note that metabolomics and other multi-omics approaches do not directly represent the organizational or regulatory structure of the IE. Rather, they provide downstream, system-level readouts, reflecting the cumulative outcomes of underlying functional processes operating across multiple levels and timescales. As such, multi-omics data are best interpreted as context-dependent profiles of IE states, useful for identifying patterns, shifts, and comparisons, but not for inferring mechanisms or organizational principles in isolation. This distinction is particularly important in educational contexts, where omics data may otherwise be misconstrued as direct mappings of physiological organization rather than as outcome-level system summaries.

Finally, systems biology modeling and network analysis provide conceptual tools for understanding how interactions between subsystems give rise to global stability and how system-level configuration constrains the admissible behavior of individual subsystems (Angione, 2019).

By making connectivity and transitions among steady states explicit, these approaches support a shift in homeostasis education from parameter-centered descriptions toward a system-level understanding of IE homeostasis, consistent with the framework developed below.

### 4.4 Expanding the scope of homeostasis or homeostatic tendency

Here, homeostasis or homeostatic tendency is expanded both in scope and in its constitutive relationship with the environment. This expansion does not impose a new conceptual structure, but reflects an interpretive realignment required to remain faithful to the biological reality that homeostasis was originally intended to capture.

#### 4.4.1 Expanding the scope

Since Cannon introduced the concept of homeostasis in 1926, it has often been interpreted in teaching as referring exclusively to normal physiological states. When disease or stress disrupt the original steady state, homeostasis is commonly described as having been “lost.” This interpretation is overly narrow. As mentioned briefly in Kuang (2023), except during rapid deterioration or recovery, living organisms typically remain in regulated, relatively stable states across a wide range of physiological, pathophysiological, subhealth, and psychological conditions. Importantly, neither Bernard nor Cannon ever stated that internal stability is restricted to normal or healthy states; rather, their analyses emphasized the persistence and regulation of the IE under challenge.

Modern metabolomics and systems-level analyses provide empirical support for this extended view (Nicholson et al., 1999). Across diverse physiological and pathological contexts, the IE, as captured by metabolomic profiles, exhibits structured and reproducible patterns rather than random disorganization. Any phenomenon describable as a *pattern* necessarily reflects a form of relative stability defined within a given temporal and spatial scale.

It is important to note that, in many physiological and pathological contexts, the apparent stability of the IE, partially reflected in selected relational parameters such as blood glucose or lipid levels, may be preserved, while the regulatory cost required to sustain that stability progressively increases. Such latent costs are not directly visible when homeostasis is represented solely in terms of stable states or setpoints. Over extended timescales, these adaptive steady states may diverge, leading to distinct long-term outcomes depending on the effectiveness of regulation.

While homeostasis or homeostatic tendency refer to the same biological reality, the former is more operational for teaching and diagrammatic representation (Figure 3; Supplementary Figure S1). By contrast, homeostatic tendency is ontological in nature: it facilitates a dynamic understanding of homeostasis and captures the directional character of the IE—that is, how successive states may be oriented and constrained over time.

#### 4.4.2 Incorporating organism–environment interplay into homeostasis

Historically, homeostasis has often been framed in an organism-centered manner in which environmental factors are acknowledged but treated primarily as external perturbations rather than as constitutive influences on internal regulation. As noted in Kuang (2023), the setpoint around which a functional parameter oscillates can instead be viewed as the outcome of a “negotiation” between the organism and its environment. Building on this earlier mention, and supported by recent studies demonstrating sustained environmental shaping of physiological regulation across contexts such as aging, climate exposure, and early-life conditions (Cole et al., 2025; Imberti et al., 2025; Zhang et al., 2025), this article treats organism–environment interplay as a constitutive component of homeostasis.

Taking Sections 3, 4 together, Figure 3 summarizes this integrative conceptual framework of IE homeostasis, illustrating how individual parameter-level regulation, the relational parameter network, hierarchical functional organization of the network, and ongoing organism–environment interaction together give rise to emergent, IE homeostasis.

For clarity, the usage of stability-related terms (e.g., parameter homeostasis, IE homeostasis, steady state, dynamic equilibrium, etc.) across organizational levels is summarized in Supplementary Table S2.

In addition, homeostasis or homeostatic tendency is distinct yet complementary to homeodynamics (Supplementary Table S3).

## 5 Pedagogical scaffolding and implementation

Given the conceptual complexity of homeostasis, effective instruction does not require immediate completeness. Rather, understanding homeostasis is best approached as a progressive process that unfolds across sustained engagement with physiology. Accordingly, the framework presented here is not intended to prescribe a fixed teaching sequence, but to provide a flexible pedagogical scaffold that can be adapted to different learner levels and curricular contexts. At early stages, instruction may emphasize the historical origin and literal meaning of homeostasis and the distinction between parameter homeostasis and IE homeostasis, with the explicit expectation that deeper understanding develops over time. More advanced concepts, including regulatory modes, layered steady states, and emergent IE homeostasis, can be introduced progressively as students’ conceptual readiness increases. A brief scaffold summarizing these teaching considerations across instructional levels is provided in Table 1.

**TABLE 1:** A progressive pedagogical scaffold for introducing homeostasis across instructional levels.

Teaching stage	Core focus	Key concepts emphasized	Explicitly deferred
Initial exposure	Conceptual orientation	Origin and literal meaning of *homeostasis*; historical context; expectation of gradual understanding	Mechanistic explanations; system-level integration
Introductory level	Parameter homeostasis	Stable or steady state oscillations as defining feature; relational nature of functional parameters	IE homeostasis; long-term adaptation
Intermediate level	Regulatory modes	Stabilizing and adapting modes at the parameter level	Layered steady states; system-level emergence
Advanced level	Relativity of homeostasis	Layered steady states (SS1, SS2); reference dependence	Full system-level abstraction
System (organism) level	Emergent IE homeostasis	IE homeostasis as an emergent property under hierarchical constraints	Reduction to isolated parameters or mechanisms

## 6 Conclusion

Building on the conceptual developments articulated by Kuang (2023), including the four dimensions of homeostasis, the notion of homeostatic tendency, the differentiation between parameter homeostasis and internal environment (IE) homeostasis, and the relativity of homeostasis, this article clarifies key limitations of parameter-centric approaches to teaching homeostasis and envisions a coherent conceptual framework for introducing IE homeostasis at the system (organism) level. The main contributions of this analysis are as follows:

- Parameter homeostasis should be described as the relatively stable oscillation of a parameter value.
- IE homeostasis is a distinct emergent property at the organism level, arising from the coordinated regulation of multiple parameters under hierarchical constraints, rather than a direct extension of individual parameter homeostasis (an integration of the homeostasis of all individual parameters).
- Modern integrative measurements, including multi-omics approaches, provide system-level readouts that support the study and teaching of IE homeostasis.
- The scope of homeostasis or homeostatic tendency is expanded in two complementary directions. First, its conceptual scope extends beyond normal physiology to include pathological, adaptive, sub-healthy, and psychological steady states. Second, organism–environment interaction is explicitly recognized as constitutive to homeostasis, rather than being treated as secondary in more traditional organism-centric formulations.
- From a pedagogical perspective, a brief scaffold is provided to facilitate teaching and learning homeostais in a progressive manner.

By clarifying how homeostasis is conceptually framed and applied, this analysis helps orient physiology education toward a more integrated understanding of regulation that brings classical physiological principles into productive dialogue with contemporary system-level perspectives and more faithfully reflects how organisms function and adapt across conditions.

## Footnotes

1 When Cannon formulated the term homeostasis, he wrote, “The term ‘equilibrium’ might be used to designate these constant conditions. That term, however, has come to have exact meaning as applied to relatively simple physico-chemical states in closed systems where known forces are balanced” (Cannon, 1926, p. 400). As long as “dynamic” is used to modify “equilibrium”, this is no longer a concern.
