# Supplementary material for: How should we teach homeostasis? Filling the gaps and envisioning the future
Source: Front Physiol. 2026 Mar 2;17:1784015. doi: 10.3389/fphys.2026.1784015 (PMC12989328; doi:10.3389/fphys.2026.1784015)
Supplement: Supplementary file 1 [file DataSheet1.docx]

**Supplemental Materials**

Serena Y. Kuang^1^, Akshata R. Naik^1^

^1^Department of Foundational Medical Studies, Oakland University William Beaumont School of Medicine, Rochester, MI 48309

Serena Y. Kuang, [kuang@oakland.edu](about:blank) | ORCID: 0000-0001-6492-6839 | Corresponding author

Top of Form

# Supplemental Figure S1

**
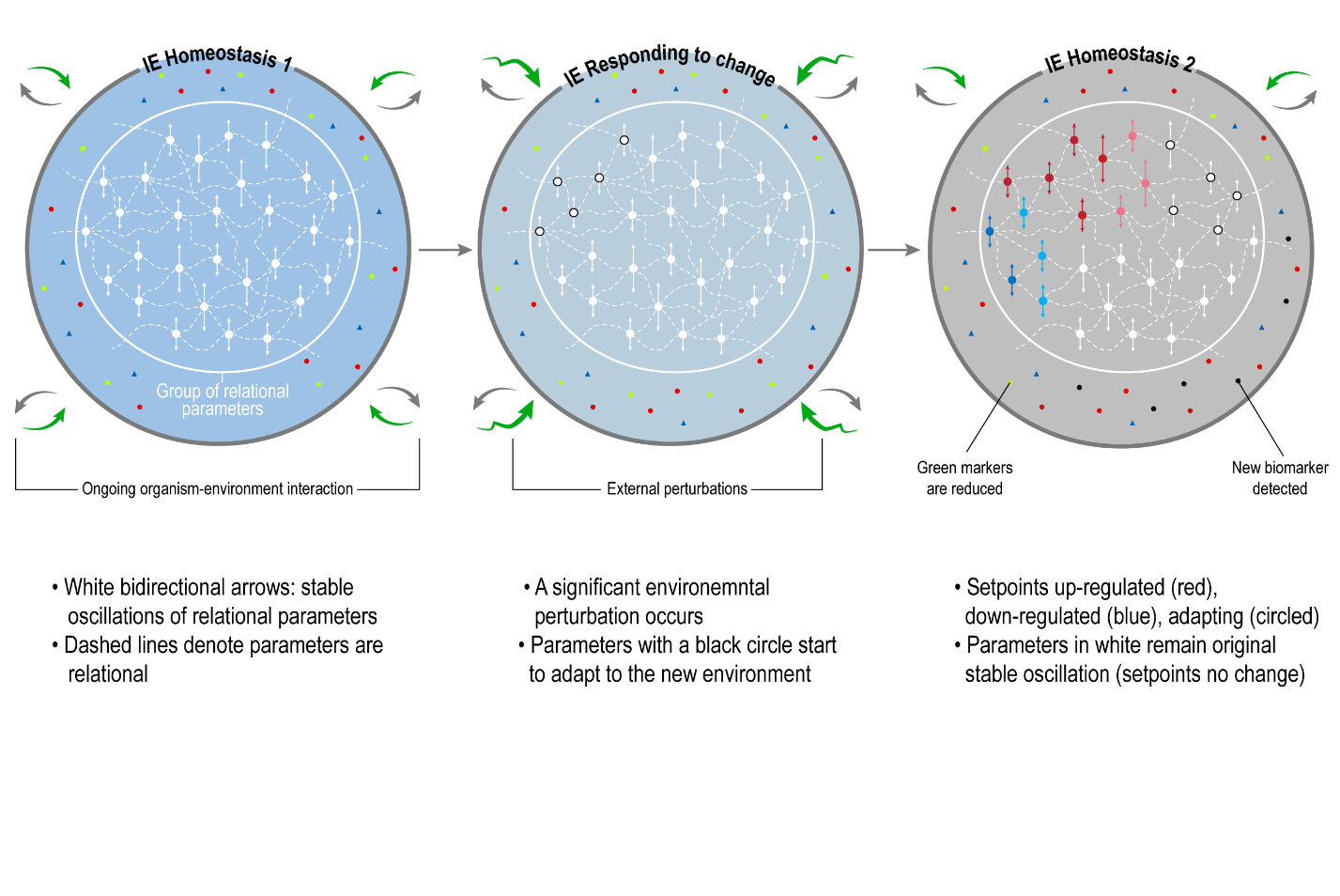
**

**Figure S1.** Qualitative illustration of key aspects of the complexity, temporality, and relativity of internal environment (IE) homeostasis. At an initial state (left, IE Homeostasis 1), parameters in the body exhibit relatively stable oscillations (illustrated using a group of sample relational parameters) and together form an integrated IE configuration under ongoing organism–environment interaction, consistent with the parameter-level regulation and relational organization depicted in Figure 3. Following a significant environmental perturbation (middle), selected parameters enter adaptive responses while others retain their original oscillatory patterns, reflecting heterogeneous yet coordinated regulation within the IE. Over extended timescales (right, IE Homeostasis 2), these coordinated changes may give rise to a new overall IE configuration, characterized by shifts in parameter oscillations and altered system-level readouts, including end metabolites detectable by metabolomics. Together, these panels emphasize the relativity of IE homeostasis: whether the IE appears stable or adaptive depends on the temporal scale and observational window. Processes that constitute adaptation over longer durations may appear as stability when examined within shorter time frames. Accordingly, IE homeostasis is best understood as a temporally framed, system-level pattern emerging from coordinated yet heterogeneous parameter-level regulation, rather than as a fixed internal state.

# Supplemental Table S2.

**Table S2.** Conceptual clarification of homeostasis-related terminology through a comparison of parameter-level homeostasis and internal environment (IE) homeostasis

| **Aspect** | **Parameter-Level Homeostasis** | **Internal Environment (IE) Homeostasis** |
| --- | --- | --- |
| **Primary object of regulation** | Individual functional parameters (e.g., blood glucose, plasma Na⁺ concentration) | The IE as an integrated relational and functional configuration emerging from coordinated subsystem dynamics |
| **Stability and steady state** | Stability is expressed as stable oscillatory patterns of individual parameters around a setpoint within a reference range; a steady state refers to pattern-level stability rather than fixed absolute values. | Stability is expressed as a coherent, higher-order pattern emerging from coordinated parameter oscillations; a steady state refers to the relative persistence of the IE despite ongoing parameter fluctuations. |
| **Dynamic equilibrium and regulation** | Dynamic equilibrium is expressed as regulated, flux-sustained stable oscillations of individual parameters, maintained by local bidirectional feedback rather than static balance. | Dynamic equilibrium refers to a persistent system-level relational pattern emerging from coordinated parameter-level regulation under continuous flux. |
| **Regulatory modes and temporal characteristics** | - Emphasizes relatively rapid, parameter-specific regulatory responses - Environmental influences are typically expressed as short-term perturbations within limited temporal windows | - Involves slower, system-level regulatory modes operating over extended time scales - Environmental conditions shape IE configuration through cumulative, long-term effects rather than immediate sensitivity |
| **Interpretive emphasis** | Focus on maintaining individual parameters within functional ranges | Focus on preserving the relational integrity of the IE as a whole |
| **Relation to the general term “homeostasis / homeostatic tendency”** | Can be described using the general term when referring to parameter-level regulation | Can be described using the general term when emphasizing emergent, system-level stability |

# Supplemental Table S3.

**Table S3.** Conceptual comparison of internal environment (IE) homeostasis/homeostatic tendency and homeodynamics as complementary observational perspectives applied to the same dynamic biological system

| **Comparison** | **Homeostasis / Homeostatic tendency** | **Homeodynamics** |
| --- | --- | --- |
| **Intellectual origin** | Coined by Walter Cannon (1926); established as a central concept in physiology; conceptually expanded in this article | Emerged gradually in systems biology, theoretical biology, and complexity science, without a single point of formal definition |
| **Observational and temporal framing** | Cross-sectional framing of internal system dynamics, describing relative stability at a given time or time scale | Longitudinal framing that connects successive system states across time, describing system evolution |
| **Explanatory emphasis** | Reciprocal emergence of relative stability and top-down constraints on subsystem dynamics | Unidirectional trajectory of system behavior |
| **Nature of stability in continuously changing systems** | Homeostasis refers to the maintenance of relative internal stability achieved through ongoing internal reconfiguration within a continuously changing system. | The term “homeo-” in homeodynamics can be understood as referring to the quasi-stationary character of system trajectories when viewed at a coarse-grained, long-term scale, rather than to the maintenance of internal stability. |

**Homeodynamics references**

Kitano, H. (2004). Biological robustness. *Nature Reviews Genetics*, **5**(11), 826–837. <https://doi.org/10.1038/nrg1471>

Paul, D. (2025). The homeodynamic theory of life: An introduction. In: *Cancer: An Integrative Approach*. Cham: Springer. <https://doi.org/10.1007/978-3-032-08808-6_8>

Trzebski, A. (1994). Homeodynamics versus homeostasis: periodicities superimposed on non-linear dynamic sympathetic tone generated in ventral medulla. *Acta Neurobiologiae Experimentalis*, **54**(2), 109–125.
